# Comparative Analysis of Cartilage Marker Gene Expression Patterns during Axolotl and *Xenopus* Limb Regeneration

**DOI:** 10.1371/journal.pone.0133375

**Published:** 2015-07-17

**Authors:** Kazumasa Mitogawa, Aki Makanae, Ayano Satoh, Akira Satoh

**Affiliations:** 1 Research Core for Interdisciplinary Sciences, Okayama University, Okayama, Japan; 2 Graduate School of Natural Science and Technology, Okayama University, Okayama, Japan; 3 Research Fellow of Japan Society for the Promotion of Science, Japan Society for the Promotion of Science, Tokyo, Japan; University of Colorado, Boulder, UNITED STATES

## Abstract

Axolotls (*Ambystoma mexicanum*) can completely regenerate lost limbs, whereas *Xenopus laevis* frogs cannot. During limb regeneration, a blastema is first formed at the amputation plane. It is thought that this regeneration blastema forms a limb by mechanisms similar to those of a developing embryonic limb bud. Furthermore, *Xenopus laevis* frogs can form a blastema after amputation; however, the blastema results in a terminal cone-shaped cartilaginous structure called a “spike.” The causes of this patterning defect in *Xenopus* frog limb regeneration were explored. We hypothesized that differences in chondrogenesis may underlie the patterning defect. Thus, we focused on chondrogenesis. Chondrogenesis marker genes, *type I* and *type II collagen*, were compared in regenerative and nonregenerative environments. There were marked differences between axolotls and *Xenopus* in the expression pattern of these chondrogenesis-associated genes. The relative deficit in the chondrogenic capacity of *Xenopus* blastema cells may account for the absence of total limb regenerative capacity.

## Introduction

Urodele amphibians, such as axolotls and newts, have great regenerative ability and can completely regenerate amputated limbs. After limb amputation, the wound is immediately covered with migrating epithelial cells that form a wound epithelium (WE) [1, 2]. WE interacts with the stump tissues, including nerves, and this interaction is considered to drive WE to form an apical epithelium cap (AEC) [3]. AEC is considered essential tissue for successful limb regeneration [4]. AEC and nerves create a regenerative environment to induce a blastema [5–10]. Once the regeneration blastema is established, molecular mechanisms similar to those during limb patterning of the developing limb bud are activated.

A few kinds of anuran amphibians, such as *Xenopus laevis*, have an intermediate limb regenerative capacity intermediate between urodele amphibians (regenerative) and amniotes (nonregenerative). The *Xenopus laevis* tadpole can regenerate a complete limb structure until stage (st.) 52; however, their regenerative capacity declines gradually thereafter [11]. After metamorphosis, *Xenopus* frog no longer retain perfect regenerative ability while they still can extend structures toward distal. The extended structure mainly consists of cartilage and cone shaped therefore called a “spike” [12–13]. The spike has neither a joint nor a branch. Moreover, neither muscles nor ossified bones develop [14–15]. Thus, *Xenopus* frog can initiate limb regeneration process but fail to form a patterned limb. Such reduced limb regeneration capability in *Xenopus laevis* can be considered to be intermediate and has been investigated to elucidate why vertebrates have lost limb regeneration ability along evolution.

To investigate amphibian limb regeneration, the accessory limb model (ALM) is a powerful experimental system in urodele amphibians [5, 16–17]. Many of recent studies were achieved using ALM in axolotls [3, 5, 17–18]. ALM is now applicable even in *Xenopus laevis* [19]. These ALM studies indicate that skin wounding in addition nerve rerouting to the wounded skin are sufficient to induce a blastema. In the axolotl ALM model, the induced ectopic blastema shows cartilage differentiation [5, 20]. However, *Xenopus* ALM blastemas do not keep growing and do not have cartilage differentiation ability [19]. However, an additional procedure, a bone wound, can confer cartilage differentiation capacity to the *Xenopus* ALM blastema. These ALM studies suggest that cartilage formation processes differ between axolotls and *Xenopus*.

Comparison of the differences in chondrogenesis during limb regeneration between regenerative (e.g., axolotl blastema) and partially regenerative (e.g., *Xenopus* frog blastema) could provide valuable insights for understanding the molecular mechanisms of limb regeneration ability. *Type I* and *type II collagen* exhibit peculiar expression patterns during chick development and newts limb regeneration [21–22]. Thus, in this study, we compared expression pattern of the cartilage marker genes during both limb regeneration and development of axolotls and *Xenopus* limbs and investigate differences in cartilage differentiation capacity between *Xenopus* and axolotl blastema cells.

## Materials and Methods

### Ethical treatment of animals

All protocols and procedures conformed to the Policy on the Care and Use of the Laboratory Animals of Okayama University. The ethics committee approved this study although any specific permission number is not assigned for our amphibian experiments. All surgery was performed under ethyl 3-aminobenzoate methanesulfonate salt anesthesia, and all efforts were made to minimize suffering.

### Animals and Surgical Procedures

*Xenopus laevis* frogs, *Xenopus laevis* tadpoles, and adult axolotls were obtained from domestic animal venders. Axolotl fertilized eggs were obtained after natural mating between adult males and females. The fertilized eggs were grown in our laboratory until they reached appropriate stages. Animals were maintained at 20–22°C in dechlorinated water. For surgical procedures, animals were anesthetized using 0.1% ethyl 3-aminobenzoate methanesulfonate salt (Sigma, MS222) pH 7.0.

All limbs were amputated at mid-zeugopod level. *Xenopus* tadpole limb buds were amputated at presumed mid-zeugopod level. *Xenopus* ALM blastemas and deep wound blastemas were induced as described previously [19]. For grafting of *Xenopus* ALM blastemas, deep wound ALM blastemas and normal blastemas, blastemas at medium bud stage were used. The blastemas were removed from the limb and mesenchymal cells isolated using forceps and scissors. The removed blastema mesenchymal tissues were nicked using a knife in order to promote PKH26 dye immersion. The PKH26 labeling procedure was as described previously [18]. After labeling, PKH26-labeled mesenchyme was washed several times with PBS and then grafted as described previously [18]. The samples were fixed on day 20 postgrafting. Three independent trials were performed for each experiment and consistent results confirmed in all cases.

### Histology

Dehydrated tissue sections were immersed in tap water to remove Optimum Cutting Temperature (OCT) Compound (Sakura Finetek), stained with Alcian blue solution (Wako) for 3 min, washed with water, stained with hematoxylin (Wako) for 5 min. washed with tap water for several minutes, stained with eosin (Wako) solution for 5 min, and finally washed with 70% ethanol. Sections were then dehydrated with ethanol and mounted using Softmount (Wako, Richmond, VA). PKH-labeled sections were stained with Alcian blue solution for 3 min, washed with water, washed with 70% ethanol, and mounted using Fluoromount (Diagnostic Bio systems).

### Reverse transcriptase polymerase chain reaction (RT-PCR)

Total RNA was isolated from regenerating blastemas using TriPure reagent (Roche). Total RNA was used as a template for first-strand cDNA synthesis using oligo (dT) primers. Primescript reverse transcriptase (TaKaRa) was used for the extension according to the manufacturer’s instructions. Each polymerase chain reaction (PCR) cycle was performed as follows: 96°C for 15 s, 58°C for 30 s, 72°C for 60 s, and a final extension for 5 min at 72°C. The following primers were used: *Xenopus type I collagen* forward, GCTGGAAAGAGTGGAGATCG; *Xenopus type I collagen* reverse, CGCTGTTCTTGCAGTGGTAA; *Xenopus type II collagen* forward, CTGGTGGTCCTGGTATTGCT; *Xenopus type II collagen* reverse, AAACCACGTTCACCTCTTGG; Axolotl *type I collagen* forward, AGGCTCCAACGAGATTGAGA; Axolotl *type I collagen* reverse, GCCCAATGCATTCTGGTAGT; Axolotl *type II collagen* forward, CACCTATGGATATTGGTGGAGC; Axolotl *type II collagen* reverse, GTACATCATCCACTTGGCTACC (As previously reported [23]).

The PCR products of those were cloned into the pTAC II vector (BioDynamics) and the plasmids were used for the probe synthesis and the following analysis (*in situ* hybridization).

### In situ hybridization

RNA *in situ* hybridization was performed on sectioned tissues. Digoxigenin (DIG)-labeled antisense RNA probes for *Xenopus type I collagen* (Gen Bank ID#: AB034701.1), *Xenopus type II collagen* (NCBI Reference Sequence ID#: NM_001087789.1), axolotl *type I collagen* (Ambystoma EST database ID#: C068652), and axolotl *type II collagen* (Ambystoma EST database ID#: C081592) were used to perform *in situ* hybridization. To synthesize antisense digoxigenin-labeled RNA probes, templates were synthesized using PCR with Ex Taq DNA polymerase (TaKaRa) and transcribed with T7 RNA polymerase (TaKaRa) and SP6 RNA polymerase (TaKaRa). Specimens were fixed overnight at RT in 4% paraformaldehyde/PBS and then decalcified in 10% ethylenediaminetetraacetic acid at room temperature (RT). Samples were treated with 5 μg/ml Proteinase K, refixed in 4% paraformaldehyde/PBS, and hybridized overnight at 63°C in a solution containing RNA probes. After hybridization, the sections were washed in 50% formamide, twice in 5× saline-sodium citrate (SSC) for 30 min at 63°C, again in 50% formamide, three times in 1.5× SSC for 30 min at 63°C, and finally in TBST (10 mM Tris–HCl, 150 mM NaCl, 0.1% Tween 20) at RT before blocking in 0.5% Blocking Reagent (Roche) for 30 min. The samples were then incubated with alkaline phosphatase (AP)-conjugated anti-digoxigenin AP antibody (Roche, 1/1,000) for 2 h at RT and then washed three times with TBST (10 min/wash). Immunolabeling was visualized using nitroblue tetrazolium chloride (Wako) and 5-bromo-4-chloro-3′- indolyl phosphatase p-toluidine salt (Wako) as a substrate for AP in buffer containing 100 mM NaCl, 100 mM Tris (pH 9.5), 20 mM MgCl_2_, and 0.1% Tween 20. Negative controls were done with the sense probes at least three times on each samples.

## Results and Discussion

### Comparison of cartilaginous gene expression patterns between axolotl limb buds, blastemas, and fracture healing cartilage

**Fig 1:**
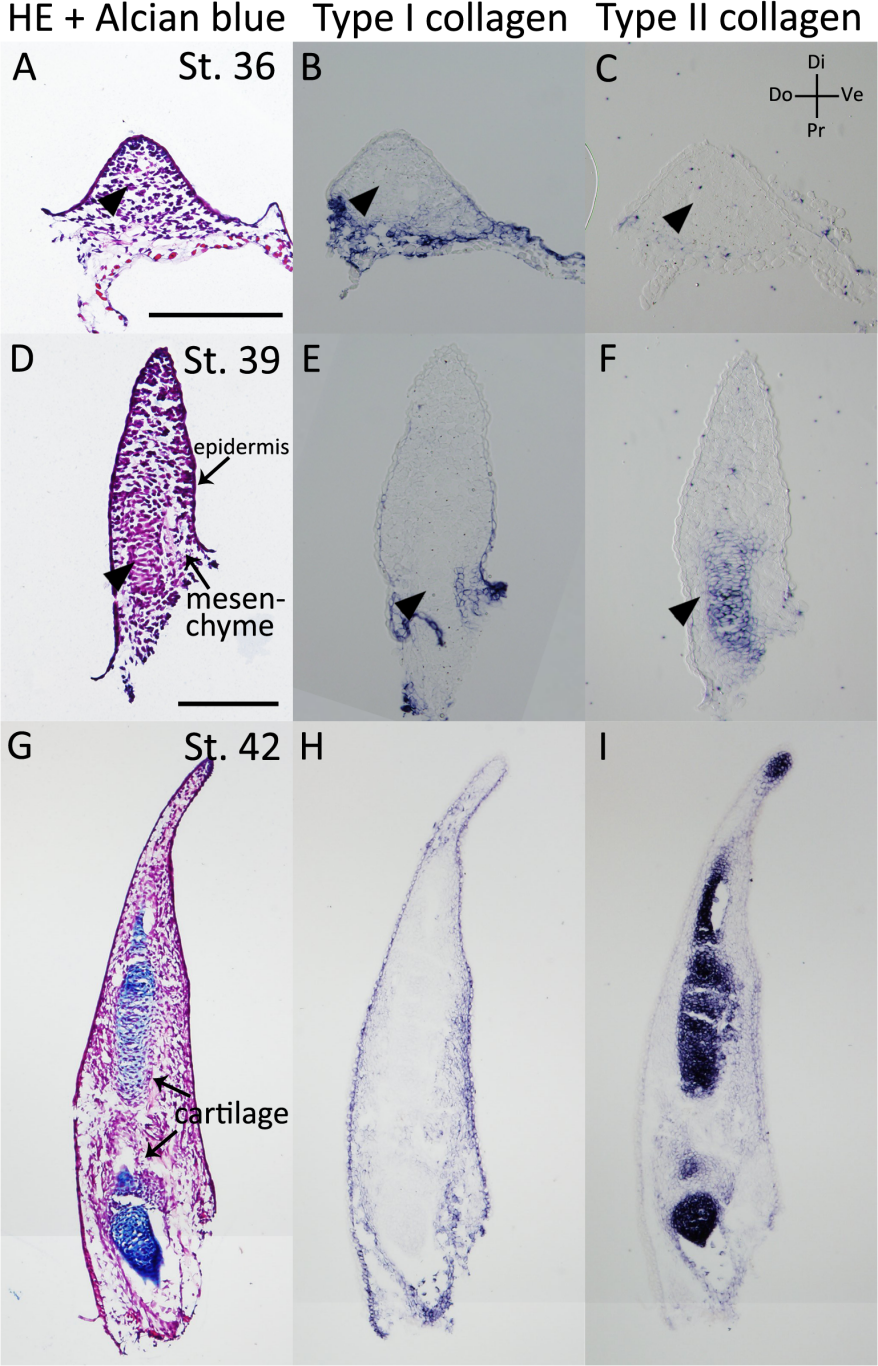
*Type I* and *type II collagen* expression patterns in the axolotl limb bud. (A-C) The stage (st.) 36 axolotl limb bud. (A) HE and Alcian blue staining. There was no Alcian blue-positive region. (B) T*ype I collagen* expression was analyzed by *in situ* hybridization. (C) *Type II collagen* expression was not observed. (D-F) The st. 39 axolotl limb bud. (D) HE and Alcian blue staining. (E) *Type I collagen* expression was observed in the dermal layer and the limb bud mesenchyme, but not in the cartilage-forming region. (F) *Type II collagen* expression was observed in the cartilaginous region. (G-I) The st. 42 axolotl limb bud. (G) HE and Alcian blue staining. (H) *Type I collagen* expression. (I) *Type II collagen* expression was observed in Alcian blue-positive cartilaginous regions. A-C are shown at same magnification. D-I are at same magnification. All scale bars are 200 μm.

We examined *type I* and *type II collagen* expression patterns in the axolotl developing limb bud (Fig 1, Table 1). Regenerating blastemas and developing limb buds are thought to use similar molecular mechanisms to develop a patterned limb [3, 24–25]. In the stage (st.) 36 and st. 39 limb buds [26–27], Alcian blue-positive cartilages were still not developed (Fig 1A and 1D). *Type I* and *type II collagen* expression patterns were examined by *in situ* hybridization, using probes designed for *alpha-1 type I collagen* and *alpha-1 type II collagen*. In the presumed cartilage region (arrowheads), neither *type I* nor *type II collagen* genes could be detected (Fig 1B and 1C). In the proximal region of the st. 39 limb bud, cartilage differentiation appeared to have started (arrowheads). *Type II collagen* was expressed prior to *type I collagen* expression (Fig 1E and 1F). In the st. 42 limb bud, Alcian blue-positive cartilage was observed (Fig 1G); although *type II collagen* was expressed (Fig 1I), *type I collagen* expression could still not yet be detected in the cartilaginous region (Fig 1H). Therefore, in a developing axolotl limb bud, *type I* and *type II collagen* demonstrate mutually exclusive expression patterns during the early phases (Table 1).

**Table 1:** *Type I* and *type II collagen* expression patterns of cartilage.

Axolotl	limb bud	blastema		fracture healing		
		distal	proximal			
*Col I*	−	−	+	+		
*Col II*	+	+	+	+		
*Xenopus*	limb bud	St.52 limb bud blastema	St. 56 limb bud blastema	frog blastema	fracture healing	deep wound ALM
*Col I*	−*	−	+	+	+	+
*Col II*	+	+	+	+	+	+

* Only in early stage.

**Fig 2:**
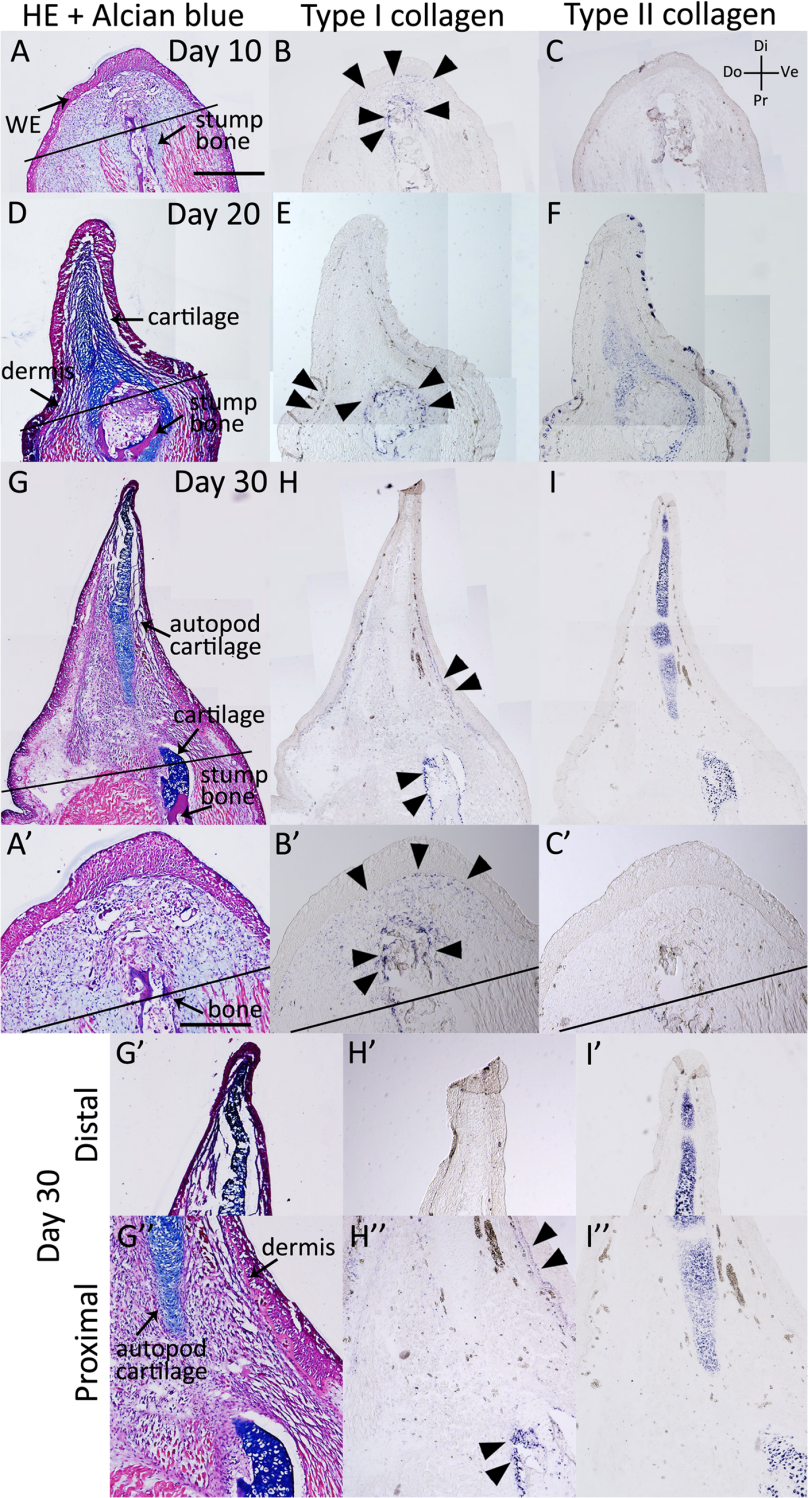
*Type I* and *type II collagen* expression patterns in the axolotl blastema. (A-C) The axolotl blastema at 10 days postamputation. (A) HE and Alcian blue staining. (B) *Type I collagen* expression was analyzed by *in situ* hybridization. *Type I collagen-*expressing cells were observed in the blastema mesenchyme and the proximal bone wound region. (C) There was no detectable *type II collagen* expression. (D-F) At 20 days postamputation. (D) HE and Alcian blue staining. (E) *Type I collagen* expression was observed in the dermal layer and the proximal bone wound region. (F) *Type II collagen* expression was observed in the Alcian blue-positive cartilaginous region. (G-I) At 30 days postamputation. (G) HE and Alcian blue staining. (H) *Type I collagen* expression was observed in the dermal layer and the proximal bone wound region. (I) *Type II collagen* expression was observed in the Alcian blue-positive cartilaginous region. A-I are shown at the same magnification. A’, B’, C’, G’, G”, H’, H”, I’ and I” are higher magnification images of A, B, C, G, H and I, respectively and A’-C’, G’-H’ and G”-H” are same magnification, Scale bar in A is 1 mm. Scale bar in A’ is 500 μm. Black bars indicate amputated lines. Black arrowheads indicate *type I collagen* expression.

Next, we examined the *type I* and *type II collagen* expression patterns in axolotl blastemas (Fig 2, Table 1). At day 10 of an axolotl blastema, *type I collagen* expression was observed in the whole blastemal mesenchyme and in particular, around the stump bone (Fig 2B), whereas *type II collagen* expression was not observed (Fig 2C). At days 20 and 30, *type I collagen* was expressed around the amputated stump bone and in the dermis (Fig 2E and 2H); however, it was not observed in the autopod cartilage of the blastema (Fig 2H). In contrast, *type II collagen* was expressed in Alcian blue-positive cartilage (Fig 2D, 2F, 2G and 2I). Thus, the axolotl blastema had *type I collagen*-negative and *type II collagen-*positive cartilage in the distal region, whereas the cartilage in the proximal region revealed both *type I* and *type II collagen* expression. This is consistent with the previous result in newt limb regeneration [22]. These results indicate that different cartilaginous gene expression patterns are observed in the proximal and distal cartilage of the axolotl blastema. The distal parts of the limb bud and blastema appear to have a similar pattern of *collagen* gene expression (Table 1).

**Fig 3:**
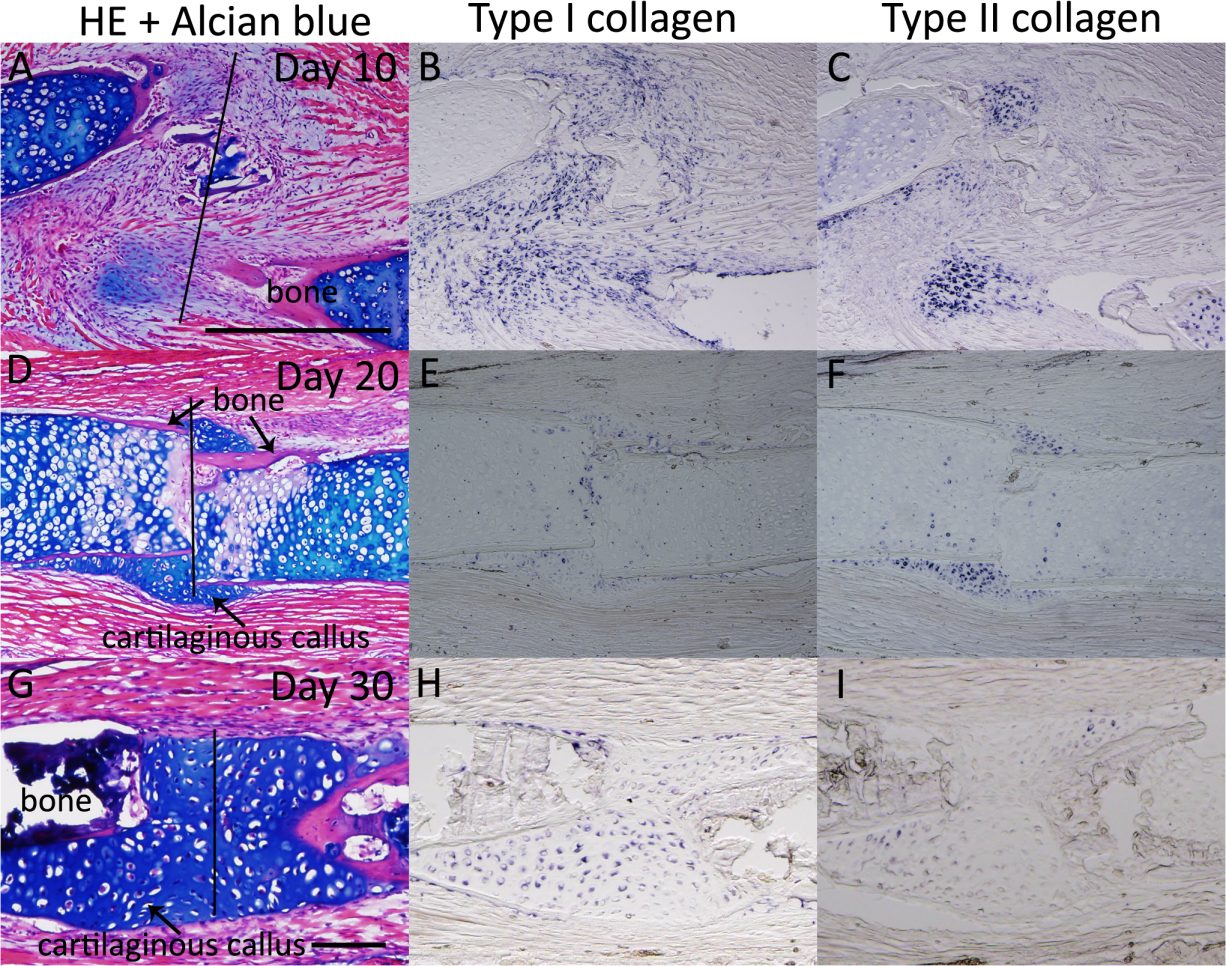
*Type I* and *type II collagen* expression patterns during bone fracture healing in axolotl. (A-C) The axolotl fracture at 10 days postwounding. (A) HE and Alcian blue staining. (B) *Type I collagen-*expressing cells were observed at the bone wound site. (C) The *type II collagen* expression area was smaller than the *type I collagen* expression area. (D-F) The axolotl fracture at 20 days postwounding. (D) HE and Alcian blue staining. A cartilaginous callus was observed in the fracture plane. (E) *Type I collagen* expression. (F) *Type II collagen* expression. (G-I) The fracture at 30 days postwounding. (G) HE and Alcian blue staining. (H) *Type I collagen* expression. (I) *Type II collagen* expression. A-F are shown at same magnification. G-I are at same magnification. Scale bars in A and G are 500 μm. Black bars indicate the bone fracture plane.

Moreover, we examined these expression patterns during axolotl fractured bone healing (Fig 3, Table 1). On day 10, both *type I* and *type II collagen* were expressed (Fig 3B and 3C). Expression of *type I collagen* was observed even in an Alcian blue-negative region (Fig 3B). On days 20 and 30, a regenerative cartilaginous callus was clearly observed expressing both *type I* and *type II collagen* (Fig 3F, 3H and 3I). These expression patterns are similar to those in the proximal part of the blastema cartilage (Table 1).

### Cartilaginous gene expression patterns in adult *Xenopus* blastema cartilage are similar to those in fractured healing cartilage

**Fig 4:**
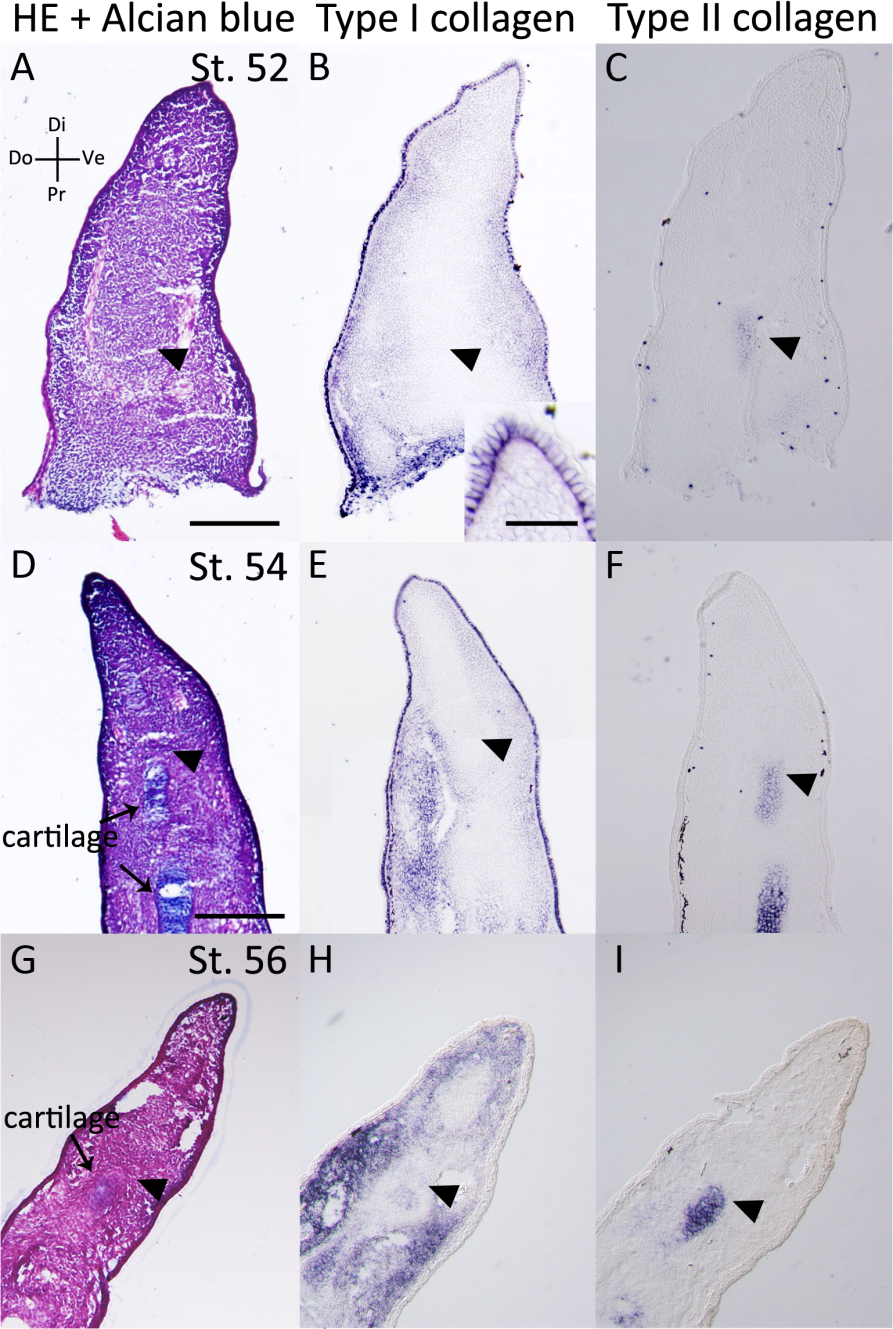
*Type I* and *type II collagen* expression patterns in the *Xenopus* limb bud. (A-C) The st. 52 *Xenopus* limb bud. (A) HE and Alcian blue staining. (B) *Type I collagen* expression. (C) *Type II collagen* expression. (D-F) The distal part of the st. 54 *Xenopus* limb bud. (D) HE and Alcian blue staining. (E) *Type I collagen* expression. (F) *Type II collagen* expression. (G-I) The distal part of the st. 56 *Xenopus* limb bud. (G) HE and Alcian blue staining. (H) *Type I collagen* expression. (I) *Type II collagen* expression. A-C are shown at same magnification. D-I are at same magnification. Scale bars in A, B insert, D are 500 μm, 200 μm, 100 μm, respectively. Arrowheads indicate presumed cartilaginous regions.

**Fig 5:**
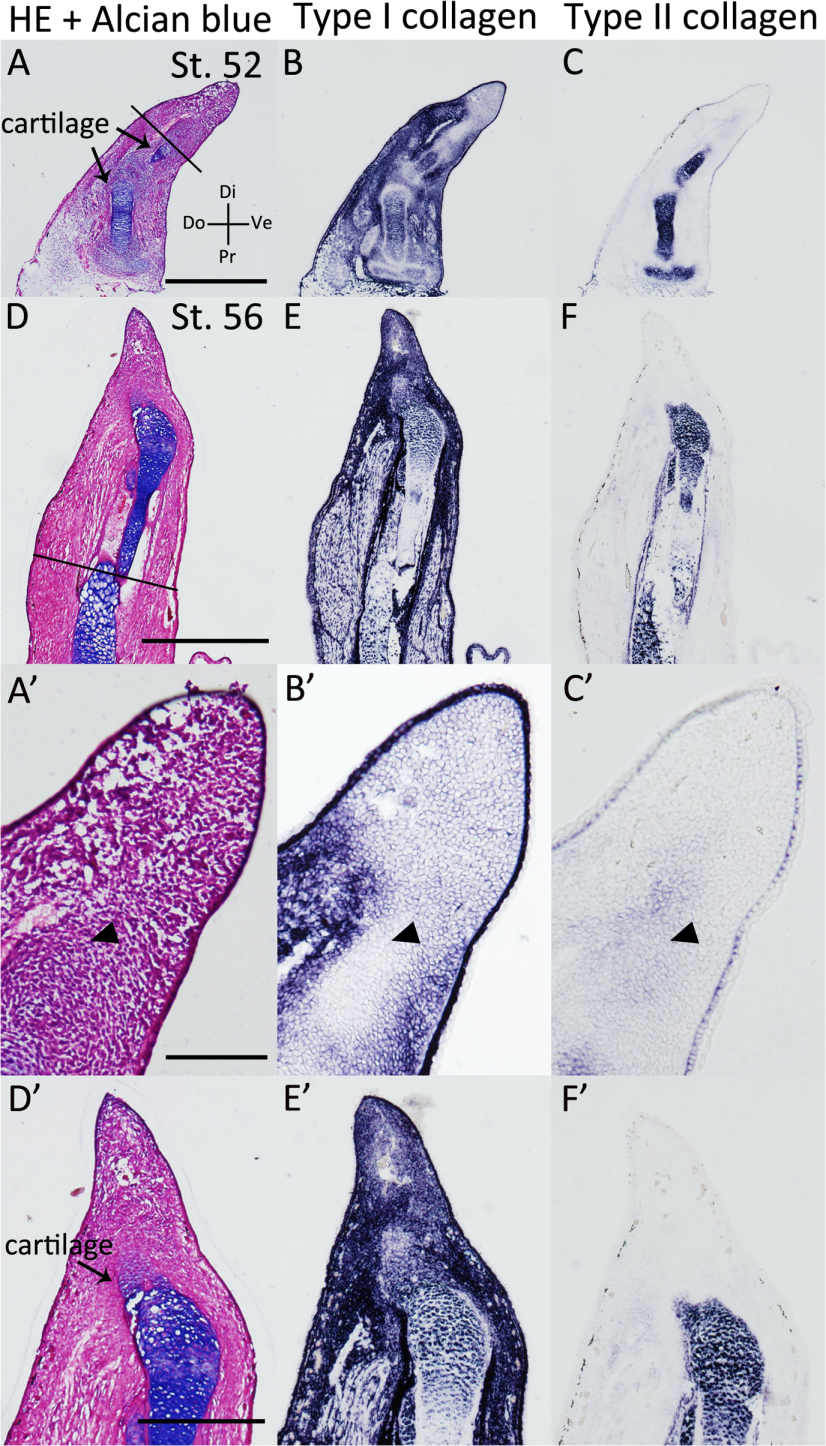
*Type I* and *Type II collagen* expression patterns in *Xenopus* stage 52 and stage 56 limb bud blastemas. (A-C) On day 10 following zeugopod amputation at st. 52 limb bud. (A) HE and Alcian blue staining. (B) *Type I collagen* expression. *Type I collagen* expression was weak in the distal region. (C) *Type II collagen* expression. (D-F) On day 10 following zeugopod amputation at st. 56 limb bud. (D) HE and Alcian blue staining. (E) *Type I collagen* expression. *Type I collagen* expression was observed throughout the entire mesenchymal region. (F) *Type II collagen* expression. A-C are shown at the same magnification. D-F are shown at the same magnification. A’-F’ are higher magnification images of A-F, respectively. Scale bars in A, D, C’, D’, are 200 μm, 500 μm, 1 mm, 250 μm, respectively. Black bars indicate amputated planes. Arrowheads indicate estimated cartilage forming areas.

**Fig 6:**
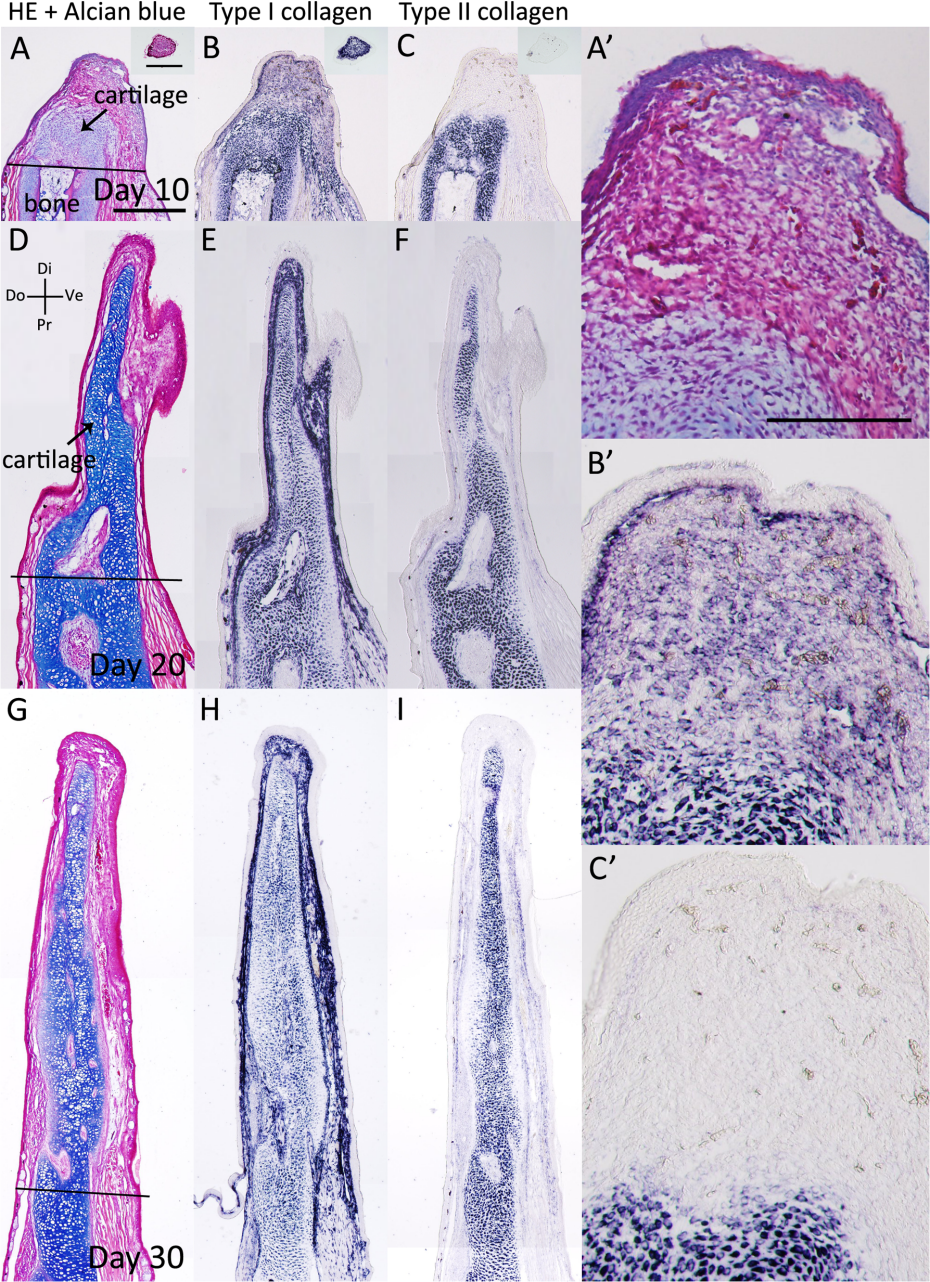
*Type I* and *Type II collagen* expression patterns in the *Xenopus* blastema. (A-C) The *Xenopus* blastema at 10 days postamputation. Insert indicates Proximal-Distal axis sections. (A) HE and Alcian blue staining. (B) *Type I collagen* expression. (C) *Type II collagen* expression. (D-F) The *Xenopus* blastema at 20 days postamputation. (D) HE and Alcian blue staining. (E) *Type I collagen* expression. (F) *Type II collagen* expression. (G-I) The *Xenopus* blastema at 30 days postamputation. (G) HE and Alcian blue staining. (H) *Type I collagen* expression. (I) *Type II collagen* expression. A-I are shown at the same magnification. A’, B’ and C’ are higher magnification images of A, B and C, respectively. Scale bars in A are 500 μm. Scale bar in A’ is 200 μm.

**Fig 7:**
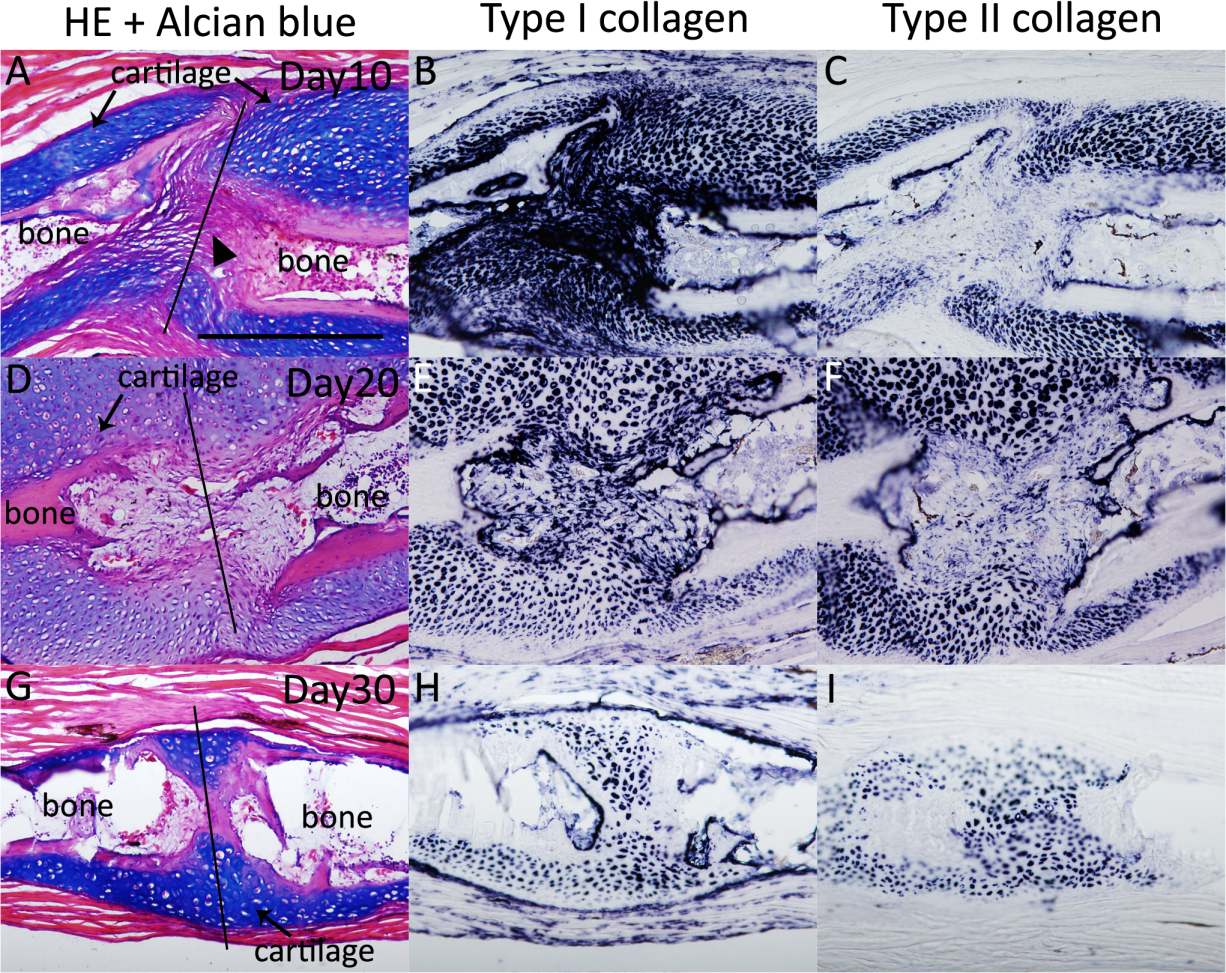
*Type I* and *type II collagen* expression patterns during *Xenopus* fracture healing. (A-C) The *Xenopus* fracture at 10 days postwounding. A cartilaginous callus was observed in the bone wound plane. (A) HE and Alcian blue staining. (B) *Type I collagen-*expressing cells were observed at the bone wound site. (C) The *type II collagen* expression area was smaller than the *type I collagen* expression area. (D-F) The *Xenopus* fracture at 20 days postwounding. (D) HE and Alcian blue staining. (E) *Type I collagen* expression. (F) *Type II collagen* expression. (G-I) The *Xenopus* fracture at 30 days postwounding. (G) HE and Alcian blue staining. (H) *Type I collagen* expression. (I) *Type II collagen* expression. All panels are shown at the same magnification. Scale bar is 200 μm. Black bars indicate amputated planes. Arrowheads indicate the gap of the amputated bone.

**Fig 8:**
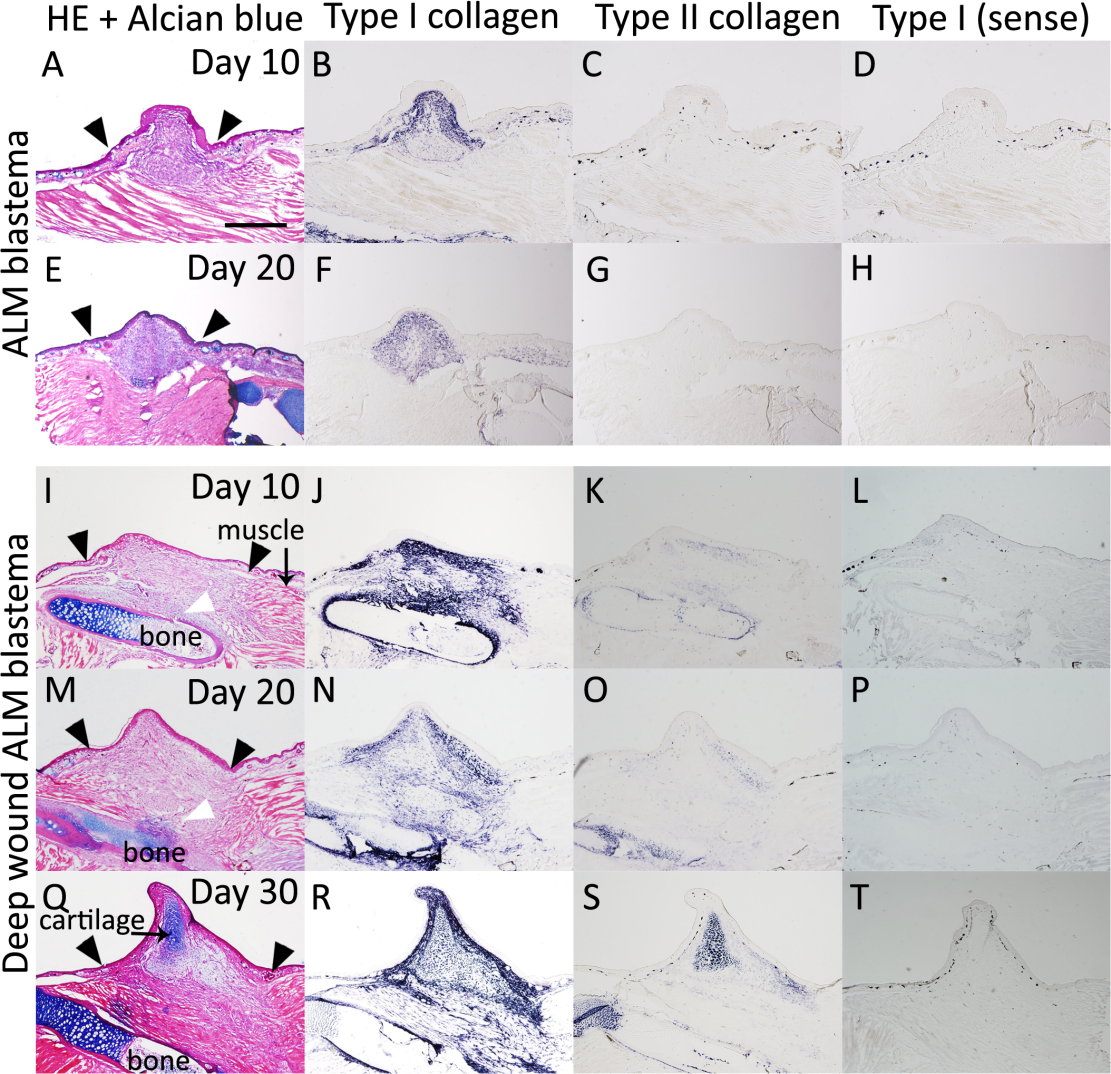
*Type I* and *type II collagen* expression patterns in *Xenopus* ALM blastemas. (A-H) The *Xenopus* ALM blastema. (A-D) The *Xenopus* ALM blastema at 10 days postoperation. (A) HE and Alcian blue staining. (B) T*ype I collagen* expression. (C) *Type II collagen* expression. (E-H) The *Xenopus* ALM with deep wound blastema at 20 days postoperation. (E) HE and Alcian blue staining. (F) T*ype I collagen* expression. (G) *Type II collagen* expression. (I-T) The *Xenopus* ALM with deep wound blastema. (I-L) The *Xenopus* ALM with deep wound blastema at 10 days postoperation. (I) HE and Alcian blue staining. (J) *Type I collagen* expression. (K) *Type II collagen* expression. (M-P) The *Xenopus* ALM with deep wound blastema at 20 days postoperation. (M) HE and Alcian blue staining. (N) T*ype I collagen* expression. (O) *Type II collagen* expression. (Q-T) The *Xenopus* ALM with deep wound blastema at 30 days postoperation. (Q) HE and Alcian blue staining. (R) *Type I collagen* expression. (S) *Type II collagen* expression. (D, H, L, P, T) Control of *in situ* hybridization experiments. Sense probe of *type I collagen*. All are shown at the same magnification. Scale bar is 500 μm. Black arrowheads indicate wound line. White arrowheads indicate bone cracked region.

We examined *type I* (*alpha-1 type I collagen*) and *type II collagen* (*alpha-1 type II collagen*) expression patterns in *Xenopus* developing limb buds, blastemas, fractured healing cartilage, and accessory limb model (ALM) blastemas (Figs 4–8, Table 1). First, we focused on developing limb buds (Fig 4). In the st. 52 limb bud [28], there was no Alcian blue-positive cartilage (Fig 4A). In the proximal region, *type I collagen* was not observed around the *type II collagen* expression area (Fig 4B and 4C). In the distal region, intense signal of *type I n*or *type II collagen* expressions were not observed in the mesenchyme (Fig 4B and 4C). However, strong signal of *type I collagen* expression was detectable in the epidermis (Fig 4B, insert). At st. 54 and st. 56, an Alcian blue-positive cartilage was observed (Fig 4D and 4G) and both *type I* and *type II collagen* were expressed in the Alcian blue-positive cartilaginous region (Fig 4E, 4F, 4H and 4I). In the distal region of the limb bud, only *type II collagen* expression was observed in the estimated cartilaginous region (Fig 4F and 4I). These results suggest that the cartilage in the limb bud expresses *type II collagen* before *type I collagen* (Table 1).

An amputated *Xenopus* limb bud completely regenerates until st. 52, following which the regenerative capacity gradually weakens. When a limb bud is amputated at st. 56, the regenerate can be recognized as a limb but is heteromorphic [11]. Postmetamorphosed *Xenopus* frogs can still initiate limb regeneration after amputation; however, they lose patterning ability, resulting in the single cone-shaped cartilage formation called a “spike.” Hence, a regeneration blastema raised in the *Xenopus* st. 56 limb bud can be considered to possess intermediate capacity (the heteromorphic shape) between adult *Xenopus* (the spike) and st. 52 limb bud (complete regeneration) [11].

Furthermore, we examined *type I* and *type II collagen* expression patterns in the regenerating *Xenopus* st. 52 and st. 56 limb buds (Fig 5, Table 1). On day 10 following zeugopod amputation at st. 52, the limb bud blastema expressed *type II collagen* in the distal region, whereas *type I collagen* expression was weak (Fig 7B and 7C). These expression patterns are similar to those in the limb bud (Fig 5, Table 1). On day 10 following zeugopod amputation at st. 56, cartilage regeneration was observed in the limb bud blastema (Fig 5D). In addition, *type I collagen* expression was observed in the distal part of the blastemal, whereas *type II collagen* expression was observed only in Alcian blue-positive cartilage (Fig 5D–5F). Cartilaginous gene expression patterns in the st. 52 limb bud blastema were similar to those in the limb bud (Fig 4, Table 1), whereas expression patterns in the st. 56 limb bud blastema were different (Table 1). Postmetamorphosed frog limb blastema (day 10 blastema) expressed *type I collagen* throughout the blastema (Fig 6B), while *type II collagen* expression was observed only near the stump bone (Fig 6C). On days 20 and 30, the expression of both *type I* and *type II collagen* were observed in Alcian blue-positive cartilage cells (Fig 6E, 6F, 6H and 6I). These expression patterns in the adult *Xenopus* blastema differ from those in the axolotl blastema (Fig 2, Table 1).

We also examined *type I* and *type II collagen* expression patterns in adult *Xenopus* fractured bone healing (Fig 7, Table 1). Cartilaginous callus formation around the fractured bone became obvious 10 days after the bone fracture (Fig 7A). *Type I* and *type II collagen* expression were observed in the forming callus, where they were recognized as Alcian blue positive (Fig 7A–7C). *Type I collagen* expression was observed in the gap of the amputated bone, while *type II collagen* expression was not observed (Fig 7B and 7C). On days 20 and 30, *type I* and *type II collagen* expression was observed around the fractured bone (Fig 7E, 7F, 7H and 7I). Regenerative blastemas (axolotl and *Xenopus* st. 52 limb bud) and limb buds (axolotl and *Xenopus* larvae) that eventually produced well-patterned structures revealed a similar sequence of *type I* and *type II collagen* expression [Col I (−) and Col II (+), Figs 1, 2, 4 and 5, Table 1]. *Type II collagen* is always expressed prior to *type I collagen* (Figs 1, 2, 4 and 5), and both are expressed in the same region (Fig 4). Such an expression sequence is not distinct from that in the developing limb bud of other vertebrates such as chicks [29]. Patterning-defective blastemas (adult *Xenopus* and *Xenopus* st. 56 limb bud) and healing bones (axolotl and adult *Xenopus*) simultaneously express *type I* and *type II collagen* from early chondrogenesis [Col I (+) and Col II (+), Figs 3, 5, 6 and 7, Table 1], distinct from regenerative blastemas and limb buds [Col I (−) and Col II (+), Figs 1, 2, 4 and 5, Table 1]. Future investigation is required to elucidate the mechanisms of the different collagen expression profiles in regeneration competent and incompetent blastemas/limb buds and what these mean.

### Cartilaginous gene expression patterns in *Xenopus* ALM blastemas

As mentioned above, the alternative experimental system ALM has been used in studying limb regeneration in urodele amphibians [3, 5, 16–17]. We previously reported that this strategy can be used to study *Xenopus* limb regeneration and that there are two types of ALM blastema in *Xenopus* [17, 19]. A *Xenopus* regeneration ALM blastema cannot keep growing and eventually disappears, whereas a *Xenopus* regeneration ALM blastema with bone damage (a deep wound ALM blastema) can keep growing and eventually form a spike [19]. We compared cartilaginous gene expressions in these two types of ALM blastemas. In the *Xenopus* ALM blastema, which cannot keep growing, only *type I collagen* expression was observed, and Alcian blue-cartilage or *type II collagen* expression was not observed (Fig 8A–8H). In the deep wound ALM blastema, *type I* and *type II collagen* expression differed (Fig 8I–8T). On day 10 and 20 after surgery, *type I collagen* expression was observed in an area where the muscle had been removed and around a cracked bone (Fig 8J and 8N), while *type II collagen* expression was only observed strongly around the cracked bone (Fig 8K and 8O). On day 30 after surgery, an Alcian blue-positive cartilaginous spike was observed with both *type I* and *type II collagen* expression (Fig 8Q–8T). These expression patterns are similar to those in the spike and the *Xenopus* fractured healing cartilage (Figs 6 and 7, Table 1).

### *Xenopus* ALM blastema cells do not have cartilage differentiation capacity

**Fig 9:**
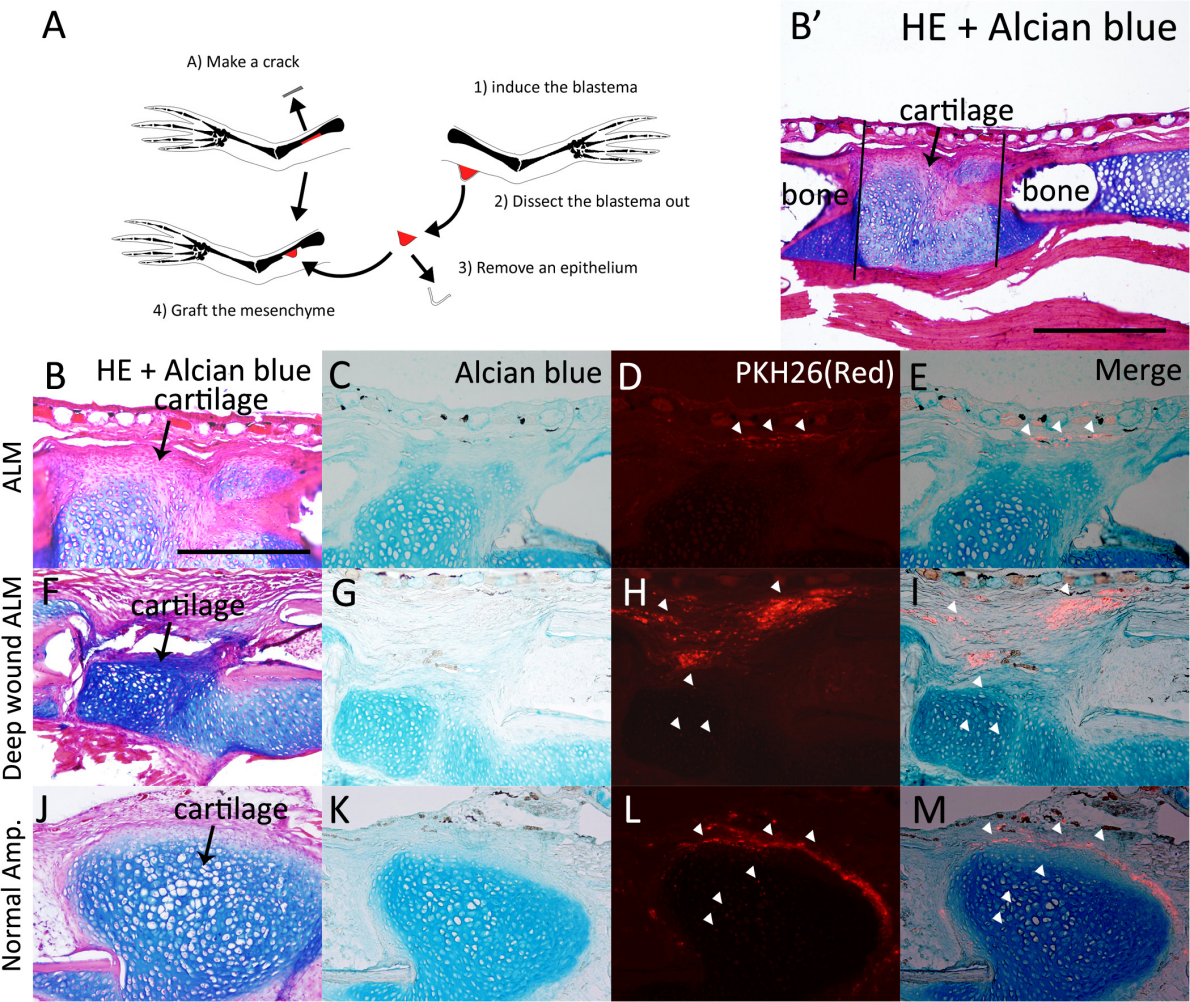
*Xenopus* ALM blastema cells do not have cartilaginous differentiation capacity. (A) The scheme of the experiment. (B-E) *Xenopus* ALM blastema cells were grafted to the bone wound site. (B) HE and Alcian blue staining. B’ is a lower magnification image of B. Black lines indicate bone crack area. (C) Alcian blue staining. The cartilaginous callus was visualized by Alcian blue stain. (D, E) Grafted cells were PKH26-positive (red). PKH26-positive cells were not observed in cartilaginous callus. White arrow heads indicate PKH26-positive cells. (F-I) Deep wound ALM blastema cells were grafted to the bone wound site. (F) HE and Alcian blue staining. (G) Alcian blue staining. (H, I) Grafted cells were observed in the cartilaginous callus. (J-M) Control experiment. Normal blastema cells were grafted to the bone wound site. (J) HE and Alcian blue staining. (K) Alcian blue staining. (L, M) Grafted cells were observed in the cartilaginous callus. B-M are shown at the same magnification. Scale bar in B is 200 μm. Scale bar in B’ is 500 μm.

Given that a *Xenopus* ALM blastema cannot keep growing and that there are different cartilaginous gene expression patterns in the two types of ALM blastema, it was suspected that a regular ALM blastema does not have cartilage differentiation capability [19]. To confirm the cartilage differentiation capacity of adult *Xenopus* frog ALM blastemas, we transplanted *Xenopus* ALM blastema cells, deep wound ALM blastema cells, or normal amputation-induced blastema cells into the bone healing regions (Fig 9). This assay has been used to test the cartilage differentiation ability of ALM blastemas in axolotls [18, 30]. Axolotl ALM blastema cells can participate in chondrogenesis in the bone healing region [18, 30]; however, clear differences could be observed with *Xenopus* ALM blastemas (Fig 9E). The induced ALM blastema was dissected and the epithelium was removed to isolate mesenchymal cells. The mesenchymal cells were labeled with PKH26 dye (red) and then grafted onto the damaged bone (Fig 9A). The damaged bone began healing and developed a cartilaginous callus (Fig 9B, 9F and 9J). If grafted *Xenopus* cells possess cartilage differentiation capacity, stained cells should be found in the cartilaginous callus, as in axolotl ALM blastema cells [18, 30]. However, blastema cells induced by ALM were not observed (Fig 9B–9E). In contrast, deep wound ALM blastema cells, which can grow spike cartilages, could be observed in the Alcian blue-positive cartilaginous callus (Fig 9F–9I). As a control, normal amputation-induced blastema cells were also transplanted in the bone healing region and grafted cells could be observed in the Alcian blue-positive cartilaginous callus (Fig 9J–9M). These results indicate that *Xenopus* ALM blastema cells do not have cartilage differentiation capacity.

The cartilage differentiation capacity of *Xenopus* ALM blastema cells clearly differs from that of axolotls (Fig 9), [5, 18–19, 30]. Normal amputation-induced and deep wound ALM blastema cells participated in bone wound healing, whereas ALM blastema cell did not (Fig 9E–9P). Normal amputation-induced blastema cells and deep wound ALM blastema cells expressed *type I collagen* before *type II collagen* (Figs 6 and 8). However, *Xenopus* ALM blastema cells do not have cartilage differentiation capacity and did not express *type II collagen*, as measured by *in situ* hybridization (Fig 9), [19]. These results imply that bone wounding is a key induction mechanism of the expression of the *type II collagen* during chondrogenesis in adult *Xenopus* frogs.

Our results may contribute to the characterization of a *Xenopus* blastema. We previously reported that a regular ALM blastema induction procedures in axolotls and *Xenopus* frogs resulted in different phenotypes [17, 19]. The induced blastema does not have cartilage differentiation capability (Fig 9). However, as the stump bone is damaged in a *Xenopus* ALM procedure, it is possible to confer an induced blastema on cartilage differentiation ability. In other words, the cartilage differentiation capacity of a *Xenopus* ALM blastema depends on bone healing responses in the stump region. Spike cartilage consistently demonstrated the same expression pattern of *type I* and *type II collagen* genes with cartilages around the healing bone (Figs 6 and 7). Hence, it is speculated that an axolotl blastema and a *Xenopus* frog blastema are substantially different (Figs 2 and 6). An axolotl blastema and a *Xenopus* tadpole st. 52 blastema, which are both regenerative blastemas, are more like limb buds. In contrast, a *Xenopus* frog blastema and a tadpole st. 56 blastema, a hypomorphic blastema, are similar to a healing bone. This may be the reason why blastema cells in *Xenopus* frogs cannot properly react to positional information, resulting in a pattern-less spike formation. Finding a way to change the cellular character of *Xenopus* frog blastema cells may provide a solution for complete limb regeneration in *Xenopus* frogs.
